# Affective temperament profiles in patients with herpes zoster and postherpetic neuralgia: a comparative analysis using TEMPS-A scale

**DOI:** 10.3389/fpsyt.2026.1741836

**Published:** 2026-02-25

**Authors:** Ümit Akkemik, Elif Göksu Yiğit Tekkanat

**Affiliations:** Department of Algology, Faculty of Medicine, Eskişehir Osmangazi University, Eskişehir, Türkiye

**Keywords:** affective temperament, herpes zoster, neuropathic pain, postherpetic neuralgia, TEMPS-A

## Abstract

**Introduction:**

This study aimed to compare affective temperament profiles between HZ and Q5 PHN patients and examine relationships among temperaments, pain intensity, quality of life, depression, and anxiety.

**Method:**

This prospective, cross-sectional study included 70 participants (35 HZ, 35 PHN) recruited from a university pain clinic. Affective temperaments were assessed using the Temperament Evaluation of Memphis, Pisa, Paris and San Diego Auto-questionnaire (TEMPS-A). Additional measures included the Hospital Anxiety and Depression Scale (HADS), Neuropathic Pain Impact on Quality-of-Life Questionnaire (NePIQoL), and Visual Analog Scale (VAS) for pain.

**Results:**

PHN patients were significantly older than HZ patients (69.31 ± 6.16 vs. 63.40 ± 9.36 years, p=0.003). Significant differences emerged across all temperament domains (p<0.001). HZ patients demonstrated predominant hyperthymic temperament (12.86 ± 3.99), while PHN patients exhibited primarily anxious temperament (16.03 ± 4.81). PHN patients showed significantly higher depressive, cyclothymic, irritable, and anxious temperament scores with large effect sizes (Cohen’s d: -0.686 to -1.456). Depression, anxiety, and quality of life measures were significantly worse in PHN patients. Strong correlations were observed between temperament dimensions and pain intensity, particularly in PHN patients (r=-0.940 to 0.934), and between temperaments and psychological measures.

**Discussion:**

Distinct affective temperament profiles differentiate acute HZ from chronic PHN patients, suggesting temperamental factors may influence pain persistence and psychological outcomes. These findings support incorporating temperament assessment into clinical evaluation for risk stratification and personalized pain management strategies.

## 1 Introduction

Herpes zoster (HZ), commonly known as shingles, is a painful neurocutaneous condition caused by reactivation of the varicella-zoster virus that remains dormant in sensory ganglia following primary varicella infection. The global incidence of HZ ranges between three and five patients per 1, 000 person-years, with approximately one million cases occurring annually in the United States (1). The incidence has been steadily increasing over recent decades, with population-based studies demonstrating a more than 4-fold increase over the last six decades (2). The varicella-zoster virus is ubiquitous and highly neurotropic, exclusively affecting humans, with the incidence of HZ increasing dramatically with age, particularly after age 50 (3).

The most significant and debilitating complication of HZ is PHN, characterized by pain persisting for three or more months following the acute HZ episode. PHN represents the most common long-term complication of varicella-zoster virus reactivation, with the risk varying significantly by age (4). The risk of developing PHN is consistently higher in elderly patients; studies have shown that PHN risk varies from 5% to more than 30%. In studies conducted on patients 50 years and older, PHN risk has generally been reported between 10-30% (5). Clinical studies have identified several independent risk factors for PHN development, including older age, presence of prodromal symptoms, greater rash severity, and more severe acute pain during the initial HZ episode (6).

The relationship between neuropathic pain conditions and psychological disturbances has gained increasing recognition in recent literature. Studies specifically examining PHN have demonstrated substantial psychological comorbidity, with one large retrospective study finding that anxiety and depression developed in 69.0% and 65.8% of PHN patients respectively (7). It is known that the prevalence of mood disorders and psychological factors is higher in patients with neuropathic pain than in patients with nociceptive pain. Furthermore, psychological factors such as catastrophizing, depression, anxiety, and stress can significantly influence pain perception (8).

Affective temperament represents a relatively stable, trait-like pattern of emotional reactivity and behavioral tendencies that exist on a continuum between normality and mood disorders. Crucially, affective temperaments are conceptualized as enduring, subclinical personality characteristics that are fundamentally distinct from current psychopathological symptoms such as major depressive episodes or acute anxiety states (9). While mood disorders represent episodic clinical manifestations, affective temperaments constitute the underlying predispositional substrate that may influence vulnerability to these conditions. Modern research confirms that affective temperaments represent the genetically determined substrate of mood disorders, with evidence suggesting they may serve as precursors to various psychiatric disorders and influence clinical dimensions such as disease course, psychopathology, and treatment adherence (10). Recent research has demonstrated the predictive role of affective temperaments in mood alterations, with specific temperament profiles predisposing individuals to the development and course of affective disorders (11). The Temperament Evaluation of Memphis, Pisa, Paris and San Diego-Auto questionnaire (TEMPS-A) is a widely validated self-report instrument developed by Akiskal and colleagues to assess fundamental affective temperament dimensions. Based on classical temperament theory rooted in the work of Kraepelin and Schneider, the instrument evaluates five distinct temperament types that represent stable emotional and behavioral patterns underlying mood disorders (12). Research applications have extended beyond psychiatric populations to include various medical conditions, where affective temperaments have been associated with pain perception, disease adaptation, and treatment outcomes, making it a valuable tool for understanding individual differences in responses to chronic medical conditions (13–15).

Although the connection between neuropathic pain and psychological factors is well-documented, and temperament is increasingly acknowledged as a stable predictor of emotional and behavioral patterns, there has been a lack of systematic studies investigating affective temperament profiles in patients with HZ and PHN. Affective temperament may impact pain perception, coping strategies, and treatment responses. Therefore, understanding these temperamental characteristics could offer important insights for clinical management and risk stratification. This study aimed to compare the prevalent profile of affective temperaments between HZ and PHN patient groups and to explore the associations among affective temperaments, pain levels, quality of life, and current levels of depression and anxiety. Given the cross-sectional nature of this study, we sought to identify correlational patterns that may inform future longitudinal research and clinical practice.

## 2 Methods

### 2.1 Study design and setting

This prospective, cross-sectional, controlled study was conducted at the Pain Medicine Division, Department of Anesthesiology and Reanimation, Faculty of Medicine, Eskişehir Osmangazi University between April 1, 2025, and September 15, 2025. The study protocol was approved by the Non-Interventional Ethics Committee of Eskişehir Osmangazi University (Date: March 27, 2025). Written informed consent was obtained from all participants. A total of 35 patients were included in each group (Group 1: HZ, Group 2: PHN).

### 2.2 Participants

Patients presenting to the pain medicine clinic with HZ/PHN diagnosis who were aged 18 years and older were considered for inclusion in the study. HZ and PHN diagnoses were made according to established clinical criteria (4). All participants needed to be able to complete all questionnaire forms included in the study and agree to participate with informed consent provided. Patients were excluded if they were under 18 years of age, had been diagnosed with fibromyalgia, cervical disc herniation, radiculopathy, myelopathy, polyneuropathy, or brachial plexopathy, or had a history of cervical vertebral trauma or fracture. Additional exclusion criteria included patients with malignancy, psychiatric illness, or cognitive impairments, pregnant and lactating patients, patients who did not complete questionnaires or declined participation, and patients diagnosed with HZ who did not attend or could not be reached for 3-month follow-up.

### 2.3 Measures

Following Data collected included age, gender, symptom duration, presence of diabetes mellitus, immunosuppression status, and VAS pain scores. Pain intensity was assessed using a Visual Analog Scale (VAS) with a 0–10 numerical rating scale, where 0 indicated “no pain” and 10 indicated “most severe pain”.

The Turkish version of the Temperament Evaluation of Memphis, Pisa, Paris and San Diego Auto-questionnaire (TEMPS-A) was used to assess affective temperament (16). The scale consists of 99 items answered with “yes” or “no” responses, evaluating five affective temperament types. Items 1–18 assess depressive temperament (18 items), items 19–37 assess cyclothymic temperament (19 items), items 38–57 assess hyperthymic temperament (20 items), items 58–75 assess irritable temperament (18 items), and items 76–99 assess anxious temperament (24 items). Each item is scored binarily (Yes = 1, No = 0).

The Turkish version of the Hospital Anxiety and Depression Scale (HADS) was used to assess anxiety and depression levels (17). This 14-item self-report questionnaire consists of two subscales: anxiety (HADS-A, 7 items) and depression (HADS-D, 7 items). Each item is scored on a 4-point Likert scale (0-3), with subscale scores ranging from 0 to 21. Higher scores indicate greater symptom severity. Scores of 8 or above on either subscale indicate clinically significant anxiety or depression.

The Neuropathic Pain Impact on Quality-of-Life Questionnaire (NePIQoL) was used to evaluate quality of life (18). This disease-specific instrument assesses five domains: physical functioning, role functioning, emotional well-being, social functioning, and cognitive functioning. The questionnaire consists of 32 items rated on a 5-point Likert scale. Total scores range from 32 to 160, with higher scores indicating poorer quality of life.

### 2.4 Statistical analysis

Descriptive statistics for continuous variables were presented as mean ± standard deviation or as median with 25th and 75th percentile values, whereas categorical variables were expressed as frequency and percentage. The distributional characteristics of the variables were assessed using the Kolmogorov–Smirnov and Shapiro–Wilk tests. Differences between two independent groups were analyzed using the Independent Samples t-test when the assumption of normality was met, and the Mann–Whitney U test was applied when this assumption was violated. For comparisons of categorical variables between groups, the Chi-square (χ²) test was used; when the expected cell frequencies were less than 5, the Fisher’s exact test was employed. Correlations between continuous variables were evaluated using the Pearson product–moment correlation coefficient for normally distributed data or the Spearman rank-order correlation coefficient for non-normally distributed data. The interpretation of correlation coefficients considered both the direction and strength of the association. To assess the magnitude of intergroup differences, effect sizes were calculated using Cohen’s d, Hedges’ g correction, and Glass’s delta, each accompanied by the corresponding 95% confidence interval. All statistical analyses were performed using IBM SPSS Statistics for Windows, Version 27.0 (IBM Corp., Armonk, NY, USA). A two-tailed p-value < 0.05 was considered statistically significant for all analyses.

## 3 Results

### 3.1 Participant characteristics

A total of 70 participants were included in this cross-sectional study, with 35 patients in Group 1 (herpes zoster) and 35 patients in Group 2 (post-herpetic neuralgia). The demographic and clinical characteristics are presented in **Table 1**. The groups were well-matched for gender distribution, with 62.9% female participants in each group (χ² = 0.000, p = 1.000). However, Group 2 was significantly older than Group 1 (69.31 ± 6.16 vs. 63.40 ± 9.36 years, p = 0.003).

**Table 1:** Demographic and clinical characteristics of study participants.

Variable	Group 1 (n=35)	Group 2 (n=35)	p-value
Age (years), mean ± SD	63.40 ± 9.36	69.31 ± 6.16	0.003*
Gender, n (%)			1.000
- Female	22 (62.9)	22 (62.9)	
- Male	13 (37.1)	13 (37.1)	
Symptom duration (days), median (IQR)	14.0 (7.0-25.5)	330.0 (195.0-525.0)	<0.001†
Diabetes mellitus, n (%)	10 (28.6)	17 (48.6)	0.086
Immunosuppression, n (%)	6 (17.1)	4 (11.4)	0.495
VAS pain score, mean ± SD	6.37 ± 1.77	7.94 ± 1.28	<0.001†

*Student’s t-test; †Mann-Whitney U test.

SD, standard deviation; IQR, interquartile range; VAS, Visual Analog Scale.

### 3.2 Affective temperaments comparison

The comparison of affective temperament scores revealed significant differences in all domains between groups (**Table 2**). Group 1 showed hyperthymic temperament as the most prominent (12.86 ± 3.99), while Group 2 demonstrated anxious temperament as the highest (16.03 ± 4.81).

**Table 2:** Comparison of affective temperament scores between groups.

TEMPS-A temperament	Group 1 (n=35)	Group 2 (n=35)	p-value	Effect Size (Cohen’s d)
Depressive	8.09 ± 3.50	12.66 ± 2.98	<0.001*	-1.406
Cyclothymic	7.46 ± 2.21	8.97 ± 2.20	0.005*	-0.686
Hyperthymic	12.86 ± 3.99	7.77 ± 2.97	<0.001*	1.447
Irritable	4.71 ± 3.30	7.94 ± 3.40	<0.001*	-0.964
Anxious	8.89 ± 5.01	16.03 ± 4.81	<0.001*	-1.456

Data presented as mean ± standard deviation.

*Student’s t-test, p<0.05.

TEMPS-A, Temperament Evaluation of Memphis; Pisa, Paris and San Diego.

### 3.3 Depression, anxiety and quality of life measures

Significant differences were observed between groups in all psychological measures (**Table 3**). Group 2 patients demonstrated significantly higher depression and anxiety scores, along with poorer quality of life compared to Group 1 patients.

**Table 3:** Comparison of depression, anxiety and quality of life between groups.

Variable	Group 1 (n=35)	Group 2 (n=35)	p-value	Effect Size (Cohen’s d)
HADS Depression	5.71 ± 2.95	9.71 ± 3.24	<0.001*	-1.292
HADS Anxiety	7.09 ± 3.44	11.71 ± 3.67	<0.001*	-1.302
NePIQoL Total Score	146.20 ± 20.92	179.29 ± 31.31	<0.001*	-1.243

Data presented as mean ± standard deviation.

*Student’s t-test, p<0.05.

HADS, Hospital Anxiety and Depression Scale; NePIQoL, Neuropathic Pain Quality of Life questionnaire.

### 3.4 Correlations between affective temperaments and pain levels

Significant correlations were observed between pain levels (VAS scores) and temperament dimensions in both groups (**Table 4**). Group 2 showed stronger correlations overall, with the strongest being between pain and hyperthymic temperament (r = -0.940) and anxious temperament (r = 0.934).

**Table 4:** Correlations between affective temperaments and pain levels.

Temperament dimension	Group 1 (n=35)	p-value	Group 2 (n=35)	p-value
	r		r	
Depressive	0.833	<0.001**	0.929	<0.001**
Cyclothymic	0.029	0.867	0.202	0.244
Hyperthymic	-0.890	<0.001**	-0.940	<0.001**
Irritable	0.617	<0.001**	0.921	<0.001**
Anxious	0.701	<0.001**	0.934	<0.001**

**p<0.01 (Spearman correlation).

### 3.5 Correlations between temperaments and psychological measures

Strong correlations were observed between temperament dimensions and depression, anxiety, and quality of life measures in both groups (**Table 5**). Hyperthymic temperament consistently showed negative correlations, while depressive, irritable, and anxious temperaments showed strong positive correlations.

**Table 5:** Correlations between temperament dimensions and psychological measures.

Temperament	HADS depression		HADS Anxiety		NePIQoL quality of life	
	Group 1	Group 2	Group 1	Group 2	Group 1	Group 2
	r	r	r	r	r	r
Depressive	0.864**	0.961**	0.876**	0.938**	0.866**	0.959**
Cyclothymic	0.037	0.231	0.176	0.065	0.157	0.137
Hyperthymic	-0.888**	-0.959**	-0.782**	-0.897**	-0.800**	-0.901**
Irritable	0.854**	0.966**	0.871**	0.956**	0.833**	0.979**
Anxious	0.877**	0.942**	0.911**	0.924**	0.882**	0.969**

**p<0.01 (Pearson correlation).

Group 1: Herpes Zoster patients; Group 2: Post-herpetic Neuralgia patients.

## 4 Discussion

The present study provides the first comprehensive analysis of affective temperament profiles in patients with HZ and PHN, revealing distinct temperamental patterns that differentiate acute from chronic neuropathic pain conditions. Our findings demonstrate significant differences in all five temperament domains between groups, with HZ patients showing predominant hyperthymic temperament while PHN patients exhibited primarily anxious temperament characteristics.

The observation that PHN patients demonstrated significantly higher levels of depressive, cyclothymic, and anxious temperaments aligns with previous research examining affective temperament in chronic pain conditions. Similar patterns have been reported in fibromyalgia patients, where depressive, anxious, and cyclothymic temperaments were significantly elevated compared to healthy controls (15). Additionally, myofascial pain syndrome patients showed comparable temperament profiles with higher depression, cyclothymic, and anxious scores (14). These consistent findings across different chronic pain conditions suggest that specific temperament patterns may represent common vulnerability factors for the development or maintenance of chronic pain states.

The relationship between temperament and chronic pain extends beyond specific neuropathic conditions to encompass broader chronic pain populations. In a comprehensive study of 207 chronic pain patients using Cloninger’s Temperament and Character Inventory, Conrad et al. found that chronic pain patients scored significantly higher on Harm Avoidance and lower on Self-Directedness and Cooperativeness compared to pain-free controls (19). This psychobiological model of personality, which distinguishes between temperament dimensions (based on neurochemical transmitters) and character dimensions (involving higher cognitive processes), provides additional insight into the mechanisms underlying chronic pain development. The finding that 41% of chronic pain patients fulfilled criteria for at least one personality disorder, with temperament dimensions predicting 23% of personality disorder symptom variance, underscores the clinical significance of temperamental assessment in pain management (19).

Studies in specific musculoskeletal conditions further support the relevance of temperamental factors in chronic pain. In knee osteoarthritis patients, depressive temperament was the most common dominant affective temperament (18.6%), followed by anxious temperament (17.4%), rates considerably higher than those observed in healthy populations (20). Importantly, this study found no association between dominant affective temperament and physical therapy outcomes, suggesting that temperamental factors may be more relevant to pain development and maintenance rather than treatment response.

The strong correlations observed between temperament dimensions and pain intensity in our study, particularly in the PHN group, echo findings from other pain-related conditions. In ankylosing spondylitis patients, positive correlations were identified between depressive and anxious temperaments and disease activity measures (13). Similarly, the relationship between anxious temperament and symptom severity has been documented in myofascial pain syndrome, where anxious temperament correlated positively with symptom severity scales (14). These parallels suggest that temperament may influence pain perception and reporting across various neuropathic and inflammatory pain conditions.

Complex regional pain syndrome (CRPS) research provides particularly compelling evidence for the role of temperament in pain persistence. In patients with CRPS secondary to tendon injury, depressive temperament was present in 41.7% of cases and anxious temperament in 16.7%, significantly higher than in non-CRPS patients. Multivariate analysis revealed that depressive and anxious temperaments were independent predictors of CRPS development, alongside nerve injury, pain intensity, and depressive symptoms (21). This study represents the first evaluation of affective temperaments in CRPS patients and demonstrates that temperamental factors may contribute to the transition from acute injury to chronic pain syndrome.

The predominance of hyperthymic temperament in HZ patients represents one of the most clinically significant findings of this study and warrants detailed consideration. Hyperthymic temperament, characterized by increased energy, optimism, reduced need for sleep, and enhanced social engagement, has been consistently associated with psychological resilience and superior emotional adaptation skills in literature (22). Individuals with hyperthymic temperament demonstrate enhanced coping capabilities, positive reframing of stressful situations, and greater capacity to maintain psychological equilibrium under adverse conditions (23). In the context of acute HZ, hyperthymic temperament may function as a protective factor through multiple mechanisms. The characteristic optimism and positive outlook may facilitate more adaptive pain appraisals, reducing catastrophizing and fear-avoidance behaviors known to promote pain chronification and higher energy levels and social engagement may prevent the withdrawal and inactivity that often accompanies acute pain, maintaining functional capacity during the healing period. The striking contrast between the hyperthymic-predominant profile in HZ patients and the anxious/depressive-predominant profile in PHN patients suggests that temperament may play a critical role in determining which patients successfully resolve acute pain versus those who transition to chronicity.

The strong associations between temperament and psychological measures observed in our study are consistent with broader research on affective temperament and mental health. In cancer patients, temperament showed indirect connections to symptoms through depression and anxiety rather than direct associations (24). Similarly, our findings demonstrate robust correlations between depressive, cyclothymic, irritable, and anxious temperaments with depression and anxiety scores, suggesting that temperament may predispose individuals to mood disorders in the context of chronic pain. This relationship has been observed across various medical conditions, including ankylosing spondylitis, where temperament characteristics were linked to psychiatric symptoms (13).

A crucial conceptual distinction must be emphasized: affective temperaments and current depressive/anxiety symptoms represent fundamentally different constructs. Affective temperaments constitute stable, trait-like predispositions representing the subclinical substrate underlying vulnerability to mood disturbances, whereas depression and anxiety scores measured by HADS reflect current, state-dependent clinical manifestations (12, 25). The predisposition model suggests that temperamental traits confer vulnerability to depression in the presence of psychosocial stressors (25). The strong correlations between temperament dimensions and HADS scores in our study likely reflect this hierarchical relationship: individuals with higher depressive and anxious temperament scores may possess an inherent susceptibility that becomes clinically apparent under chronic pain stress. Conversely, hyperthymic temperament has been consistently shown to have a uniquely protective effect against mood and anxiety disorders (26) and demonstrates a strong relationship with psychological resilience (22). This trait-state distinction has important clinical implications for pain management.

The clinical utility of temperament assessment extends beyond diagnostic considerations to treatment planning. The Temperament and Character Inventory has shown promise in identifying core personality disorder features in chronic pain patients, with Self-Directedness and Cooperativeness correctly classifying 75.8% of patients regarding personality disorder presence (19). Moreover, temperament assessment may guide therapeutic interventions, as Character Dimensions are responsive to cognitive-behavioral therapy while Temperament Dimensions may require pharmacological approaches.

The clinical significance of our findings is underscored by the large effect sizes observed for temperament differences between groups, particularly for anxious (d = -1.456) and depressive (d = -1.406) temperaments. These effect sizes exceed those typically reported in personality research and suggest clinically meaningful differences. In surgical populations, temperament assessment has proven valuable for predicting treatment outcomes, with depressive and anxious temperaments negatively affecting recovery following rotator cuff surgery (27). This supports the potential utility of temperament assessment in identifying patients at risk for poor outcomes in the transition from acute to chronic pain.

However, our findings differ from some studies that found no significant temperament differences between patient groups and controls. In a large cancer patient cohort, no cancer-specific temperament profile was identified when compared to general population samples (24). This discrepancy may reflect the different nature of neuropathic pain conditions compared to cancer, where pain is often secondary to treatment or disease progression rather than representing the primary pathological process.

The strong correlations between temperament and quality of life in our study align with research in fibromyalgia, where temperament characteristics were significantly associated with disease severity (15). In myofascial pain syndrome, negative correlations were found between physical and mental health scores and specific temperament dimensions (14). Our findings demonstrate similarly robust associations, with depressive, anxious, and irritable temperaments showing strong positive correlations with poorer NePIQoL scores (r = 0.959-0.979 in PHN group), while hyperthymic temperament showed a protective effect (r = -0.901). These consistent findings across pain conditions suggest that temperament assessment may provide valuable insights into patient-reported outcomes and functional impairment.

Several methodological limitations should be acknowledged. The cross-sectional design precludes causal inferences regarding the directionality between temperament and chronic pain. We cannot determine whether temperamental patterns predispose individuals to chronic pain or whether chronic pain influences temperament expression. The relatively modest sample size and potential selection bias may limit generalizability to more diverse populations. The absence of a healthy control group limits contextualization of observed temperamental patterns relative to pain-free individuals.

The high comorbidity between chronic neuropathic pain and psychiatric disorders presents methodological challenges. While we employed statistical controls, completely isolating temperamental contributions from concurrent mood and anxiety states remains difficult. Self-report instruments may introduce response bias, though such measures are widely validated in temperament research. Our study did not assess temperamental stability over time or comprehensively evaluate other relevant psychological constructs such as coping strategies and social support. We also did not account for potentially important confounding variables including treatment modalities or socioeconomic factors.

An important methodological consideration is the significant age difference between groups (69.31 ± 6.16 vs. 63.40 ± 9.36 years, p = 0.003). This disparity is clinically expected, as older age is a well-established risk factor for PHN, with relative risk estimates per 10-year increase ranging from 1.22 to 3.11 (28). However, age may represent a potential confounding factor in the interpretation of temperament differences. Although affective temperaments measured by TEMPS-A demonstrate good long-term stability over time (29), the literature also indicates that temperament scores may show some sensitivity to demographic and clinical variables, including age (30). Therefore, while the observed temperament differences appear robust, the potential contribution of age-related influences cannot be entirely excluded. Future studies using age-matched designs would help to clarify the extent to which these findings are independent of age effects.

Several correlations in the PHN group were exceptionally high (r > 0.90), particularly between temperament dimensions and pain intensity. While these strong associations underscore a robust temperament–pain relationship, methodological factors may have contributed. The modest sample size (n= 35 per group) may have inflated correlation estimates, and the exclusive use of self-report instruments may have introduced common method variance. Future research with larger samples and multi-method approaches would help establish more precise estimates.

## 5 Conclusions

This study demonstrates that distinct affective temperament profiles differentiate patients with acute herpes zoster from those with chronic post-herpetic neuralgia. PHN patients exhibited significantly elevated depressive, cyclothymic, irritable, and anxious temperaments, while HZ patients showed predominant hyperthymic temperament characteristics. The strong correlations between temperament dimensions and pain intensity, depression, anxiety, and quality of life suggest that affective temperament plays a significant role in the transition from acute to chronic pain and psychological adaptation. These findings support the integration of temperament assessment into clinical evaluation for risk stratification and development of personalized pain management strategies. Future longitudinal research with larger samples is needed to establish causal relationships and evaluate the utility of temperament-based interventions in preventing the transition to chronicity and improving outcomes in patients with neuropathic pain conditions.

## Data Availability

The raw data supporting the conclusions of this article will be made available by the authors, without undue reservation.
